# A Multi-Mode Bioactive Agent Isolated From *Ficus microcarpa* L. Fill. With Therapeutic Potential for Type 2 Diabetes Mellitus

**DOI:** 10.3389/fphar.2018.01376

**Published:** 2018-11-27

**Authors:** Nosheen Akhtar, Laila Jafri, Brian D Green, Saima Kalsoom, Bushra Mirza

**Affiliations:** ^1^Department of Molecular Medicine, National University of Medical Sciences, Rawalpindi, Pakistan; ^2^Department of Biochemistry, Bahauddin Zakariya University, Multan, Pakistan; ^3^Advanced ASSET Centre, Institute for Global Food Security, School of Biological Sciences, Queen’s University Belfast, Belfast, United Kingdom; ^4^Pakistan Institute of Engineering and Applied Sciences, Nilore, Pakistan; ^5^Department of Biochemistry, Quaid-i-Azam University, Islamabad, Pakistan

**Keywords:** α-glucosidase, α-amylase, dipeptidyl peptidase 4, *Ficus microcarpa*, Plectranthoic acid, AMPK

## Abstract

Type 2 diabetes is a metabolic disorder, characterized by hyperglycemia and glucose intolerance. Natural products and its derived active compounds may be achievable alternatives for the treatment of type 2 diabetes. In present study we investigated the antidiabetic potential of *Ficus microcarpa* and isolated bioactive compounds i.e., Plectranthoic acid (PA) and 3,4,5,7-Flavantetrol (FL). Anti-hyperglycemic potential was evaluated *via* α-glucosidase, α-amylase and dipeptidyl peptidase 4 (DPP-4) assays. 5’AMP-activated kinase (AMPK) activation potential was assessed by using primary hepatocytes. Distribution of PA in different parts of *Ficus microcarpa* was evaluated by using rapid high-performance liquid chromatography (HPLC). Ethyl acetate fraction (FME) exhibited significant inhibition of α-glucosidase, α-amylase, and DPP-4, therefore, was selected for isolation of bioactive compounds. Among isolated compounds PA was more potent and possessed pleotropic inhibitory activity with IC_50_ values of 39.5, 55.5, and 51.4 μM against α-glucosidase, α-amylase, and DPP-4, respectively. Our results showed that PA is also a potent activator of AMPK which is a central hub of metabolic regulation. Molecular docking studies confirmed the activity of PA against α-glucosidase, α-amylase, and DPP-4. Rapid HPLC method revealed that maximum concentration of PA is present in the stem (2.25 μg/mg dry weight) of *Ficus microcarpa*. Both *in vitro* and *in silico* studies proposed that *Ficus microcarpa* and its isolated compound PA could be an important natural source for alleviating the symptoms of type 2 diabetes mellitus and we suggest that PA should be explored further for its ultimate use for the treatment of type 2 diabetes.

## Introduction

Diabetes mellitus (DM) is multifactorial disorder of metabolism which is described by high blood glucose levels known as hyperglycemia (Hu et al., 2013). Increase in blood glucose level results in non-enzymatic glycosylation of numerous proteins and thus leads to chronic complications of DM such as nephropathy, retinopathy and neuropathy (Lebovitz, 2001). Type 2 diabetes mellitus (T2DM) leads to hyperglycemia, one of the reasons is insufficient insulin production. Post-prandial hyperglycemia is a key feature of T2DM and several therapies focus on minimizing glycaemic excursions after a meal. For example, if α-glucosidase and α-amylase are inhibited it will reduce carbohydrate digestion in the lumen of the gut and, thus, minimizing the amount of glucose available for absorption thereby curbing post-prandial hyperglycemia (Bhandari et al., 2008). Another enzyme that can be targeted to minimize post-prandial hyperglycemia is dipeptidyl peptidase-4 (DPP-4) which is serine protease. DPP-4 is famous for inhibition of two incretin hormones. One of them is glucagon-like peptide 1 (GLP-1) and other is glucose dependent-insulinotropic polypeptide (GIP; Deacon et al., 1995). These incretin hormones are main stimulators of post-prandial insulin excretion, thus play role in the regulation of glucose levels after taking meal (Green et al., 2004). Inhibition of DPP-4 will result in enhancement of activity of incretin hormone. If DPP-4 is blocked GLP-1 and GIP will not undergo degradation and their half-lives will be improved and, consequently, will leads to the improvement of glucose metabolism. The risk of hypoglycemia will also be reduced (Lambeir et al., 2003).

Representatives of clinical antidiabetic drugs which inhibit α-glucosidase /α-amylase include voglibose, miglitol, and acarbose. Vildagliptin, sitagliptin, and saxagliptin are examples of clinical DPP-4 inhibitors. Some adverse effects of these drugs, like hypoglycemia, weight gain, fluid retention, congestive heart failure, fractures, abdominal discomfort, increased intestinal gas, and diarrhea, are reported (Etxeberria et al., 2012). Therefore, there is a continuous interest in the discovery of naturally occurring inhibitors of α-glucosidase, α-amylase and DPP-4, which hopefully are non-toxic, lower cost and have less adverse effects (Ren et al., 2011).

Reports show that the T2DM is also accompanied by decline in 5′ adenosine monophosphate-activated protein kinase (AMPK) activity. The AMPK sense the metabolic status of the cell. If the cell is in stress, the ratio of ADP: ATP and/or AMP: ATP will be increased and AMPK will be activated. On activation, AMPK stimulate catabolic pathways and switch off anabolic pathways. AMPK on activation will also inhibit cell-cycle thus regulating glucose levels, lipid and protein metabolism. AMPK activators are reported to be valuable for the treatment and/or prevention of T2DM (Ruderman and Prentki, 2004). Many compounds activate AMPK such as phenformin, buformin, AICAR, and some non-steroidal anti-inflammatory drugs (NSAIDs; Pollak, 2012). Natural flavonoids, polyphenols, anthocyanin, and berberine are also been presented to trigger AMPK (Hwang et al., 2007). Although several AMPK stimulators have been identified, each has its own limitations. AICAR has a short half-life and poor bioavailability, phenformin and buformin induce lactic acidosis, and metformin may increase the risk of death in non-obese individuals. These are also recognized to have many targets and toxic effects which are not dependent on AMPK. So, need of the hour is to explore more AMPK activators for the treatment of T2DM.

Various types of plants have been used for several centuries, worldwide, not only as dietary supplements but also as traditional treatment regimens for many diseases. *Ficus microcarpa* is one of the Ficus species which is potentially valuable for its medicinal usage, but is also a food ingredient (e.g., Okinawa noodles, a traditional food in Japan). Anti-diabetic activity has previously been reported for *Ficus microcarpa* extracts (Lakshmi et al., 2010), but no studies have isolated or identified anti-diabetic compounds from this plant. The aim of the present study was to perform the bioactivity guided isolation on *Ficus microcarpa* to identify potential compounds with inhibitory activity against α-glucosidase, α-amylase, and DPP-4. We report for the first time that *Ficus microcarpa* and Plectranthoic acid (PA) contains significant inhibitory activity against the above mentioned enzymes and that PA (isolated for the first time from this plant) could have potential for the treatment of T2DM. Previously, we reported the anti-cancer potential of PA and also showed that PA is non-toxic to normal cells (Akhtar et al., 2016). Many reports show that the drugs with antidiabetic potential increase AMPK activity. We already reported the AMPK activation potential of PA using cancer cell lines but in this study we evaluated the AMPK activation potential of PA using hepatocytes. Furthermore, using high performance liquid chromatography coupled with diode array detector (HPLC-DAD), we have identified the specific parts of *Ficus microcarpa* which are rich in PA.

## Materials and Methods

### Plant Material

Fresh aerial parts of *Ficus microcarpa* were collected and identified, as already reported (Akhtar et al., 2015). Voucher specimen (33) accession number 128084 was deposited in Herbarium of Medicinal Plants of Pakistan, Quaid-i-Azam University, Islamabad 45320, Pakistan.

### Extraction and Fractionation

**FIGURE 1:**
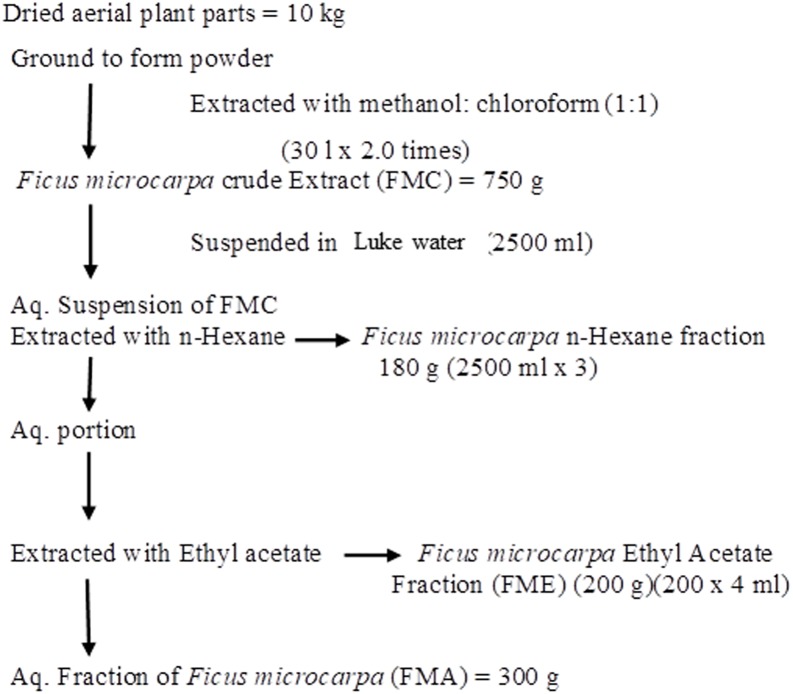
Schematic representations of preparation of crude extract of *Ficus microcarpa* (FMC) and its fractionations. FMN; n-Hexane fraction, FME; Ethyl acetate fraction, FMA; Aqueous fraction.

Aerial parts of *Ficus microcarpa* (17 kg) were dried and extracted using solvent system (Methanol/Chloroform; 1:1) following the procedure reported by Jafri et al. (2017). We obtained almost 10 kg dry weight (DW) which was grounded to fine powder. Scheme followed for preparation of crude extract (FMC) and fractions, i.e., n-hexane fraction (FMN), ethyl acetate fraction (FME) and aqueous fraction (FMA), is presented in Figure 1.

### Isolation of Bioactive Compounds

For isolation of compounds with anti-hyperglycemic potential, FME (200 g) was selected, due to better α-glucosidase, α-amylase and DPP-4 inhibition activities. FME was further fractionated by silica gel chromatography using n-hexane, ethyl acetate (EA) and methanol (MeOH) step gradient system with gradually increasing polarity, as reported by Jafri et al. (2017). Briefly, FME was dissolved in appropriate solvents and were adsorbed on the silica gel 60 (70–230 mesh, Merck, Germany) in the ratio of 1 g sample on 1 g silica and dried in a fume hood. Then a glass column was loaded with 1000 g silica gel 60 (230–400 mesh, Merck, Germany) and the dried sample was loaded on the top. A protective layer (2 cm) of silica gel was also added after loading the sample. The column was eluted with gradient change in mobile phase. Each fraction of 200 ml was collected and dried in rotary evaporator. A total of 150 subfractions were obtained and those possessing similar TLC profile, based on R*f* values, were combined together to obtain 6 major subfractions i.e., *Ficus microcarpa* ethyl acetate fraction A (FMEA), *Ficus microcarpa* ethyl acetate fraction B (FMEB), *Ficus microcarpa* ethyl acetate fraction C (FMEC), *Ficus microcarpa* ethyl acetate fraction D (FMED), *Ficus microcarpa* ethyl acetate fraction E (FMEE) and *Ficus microcarpa* ethyl acetate fraction F (FMEF).

**FIGURE 2:**
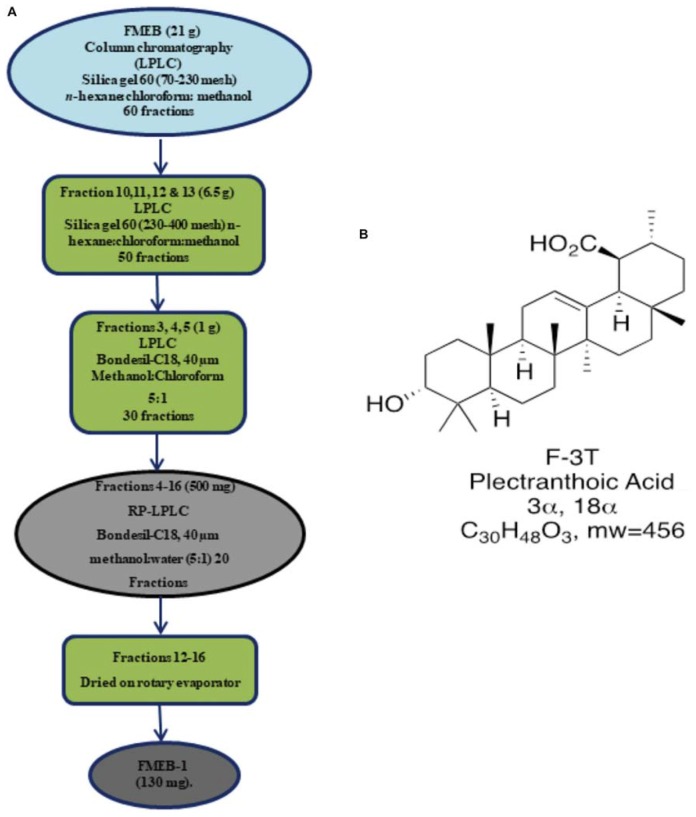
**(A)** Schematic demonstration of isolation and purification of *Ficus microcarpa* ethyl acetate fraction B (FMEB-1). FME; Ethyl acetate fraction of *Ficus microcarpa* **(B)** Molecular structure of FMEB-1, identified as Plectranthoic acid (PA).

**FIGURE 3:**
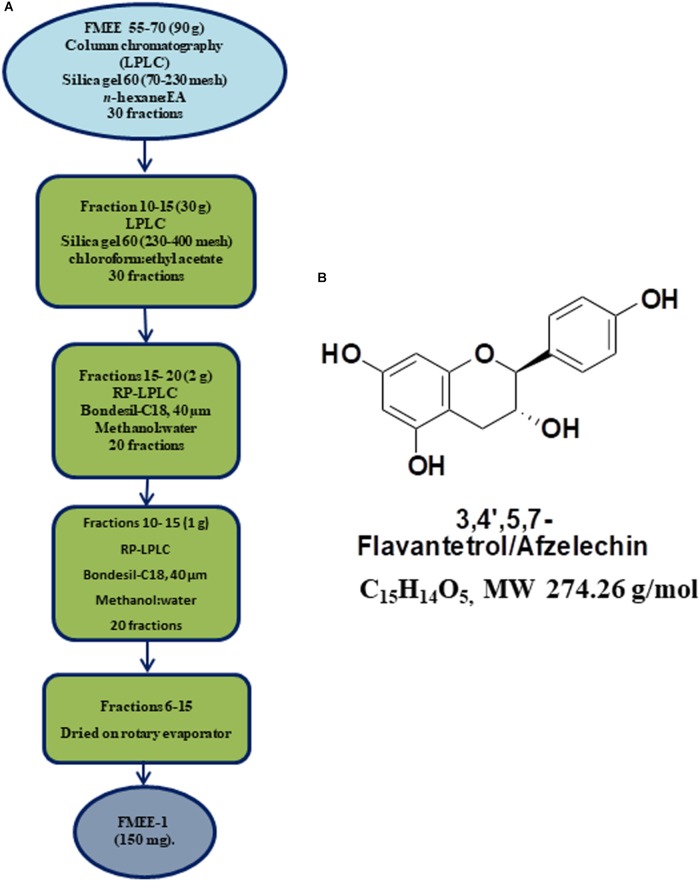
**(A)** Schematic demonstration of isolation and purification of *Ficus microcarpa* ethyl acetate fraction E (FMEE-1). FME; Ethyl acetate fraction of *Ficus microcarpa* **(B)** Molecular structure of 3,4,5,7-Flavantetrol (FL).

Flash column chromatography (silica gel 60; Merck) was performed for further fractionation. Out of 6 subfractions, FMEB, formed by combining fraction 10–20, was subjected to column chromatography (silica gel 60, 5–40 μm), using mobile phase from n-hexane to methanol in a gradient manner, and pure compound FMEB-1 was isolated, as shown in Figure 2A. FMEE (90 g combined fraction 55–70 from gravity column) was also subjected to column chromatography and other pure compound FMEE-1 was obtained, as shown in Figure 3A.

### Structural Elucidation of Isolated Compounds

A combination of mass spectrometry and nuclear magnetic resonance spectroscopy (NMR) spectroscopy (Bruker AVANCE (400 MHz NMR) was used to identify isolated compounds. Both one dimensional and two dimensional NMRs were carried out: ^1^H, ^13^C, ^1^H-^13^C heteronuclear single quantum correlation (HSQC),^1^H-^13^C heteronuclear multiple bond correlation (HMBC), ^1^H-^1^H correlation spectroscopy (COSY) and nuclear overhauser effect spectroscopy (NOESY). Spectra of pure compounds were processed by using Bruker 1D-NMR and 2D-NMR software. The structures were confirmed by comparison of spectroscopic data with reference data from available literature. NMR data of PA is shown in Supplementary Figure 1.

Plectranthoic acid (PA): (20 S) 3α-hydroxy-18 α, 19 α H-urs-12-en- 30β-oic acid, colorless needles, m.p. 260,” [α] D25”+59° (c 0.1, MeOH), M+ at m/e 456.3628. IR: max cm-1 3425 (-OH), 2930, 3200-2500 (br, -COOH), 1695, 1680, 1380, 1360. 1020. 820. H-NMR 1220 MHz). δ 0.68 (3H. s. C-24Me). 0.72 i3H, C-25 Me), 0.8 and 0.90 (9H;.s, C-23, c-26, c-27 Me’s), 0.96 (3H, s, C-28 Me), 1.05 (3H, C-29 Me), I.3Gl.80 (l8H, m),2.10 (IH, d, *J* = 5.2 Hz), 2.90 (IH, t, *J* = 16.9, 4 Hz), 4.37 (IH, m, W1/2 16.8 Hz), 5.12 (lH, s, br). MS: 456 (M+), 438 (M+-HzO), 41 I, 367,366,248 (100%), 208,207,204,203, 189. The NMR structure revealed that the isolated compound is PA, as shown in Figure 2B. The data was consistent to the reported literature (Razdan et al., 1982).

3,4,5,7- (FL): Description Computed from Structure Canonical SMILES: C1C(C(OC2 = CC( = CC( = C21)O)O) C3 = CC = C(C = C3)O)O. InChI: InChI = 1/C15H14O5/c16-9-3-1-8(2-4-9)15-13(19)7-11-12(18)5-10(17)6-14(11)20-15/h1-6,13,15-19H,7H2. The molecular structure of FL is shown in Figure 3B. NMR data of FL is presented in Supplementary Figure 2.

### α-Glucosidase Inhibition Assay

α-Glucosidase inhibition activity of the fractions and pure compounds (both PA and FL) was determined using the method of Li et al. (2005), while few modifications were made, according to system suitability. In this assay 4-nitrophenyl β-D-glucopyranoside (pNPG) was used as a substrate and enzymatic cleavage resulted in production of p-nitrophenol (yellow color), monitored at 405 nm using microplate reader (Biotec Elx-800, United States). The decrease in absorption spectrum of reaction mixture indicated the inhibition of α-glucosidase activity of the sample under test. Assays were conducted in triplicate in 96-well microtitre plates. For crude extract and fractions the final concentrations used were 200, 66.6, 22.2 and 7.4 μg/ml while for pure compounds the concentrations used were 100, 50, 25, 12.5, 6.5 μM. Acarbose was used as positive control while DMSO was used as negative control. Percentage of inhibition was calculate by using following formula.

(1) Inhibitory activity=(OD control−OD test sample)/OD control×100

IC_50_ was calculated by using table curve ASIN software (2D v4) through linear regression analysis.

### α-Amylase Inhibition Assay

Crude extract of *Ficus microcarpa*, its fractions and pure compound (PA) were then subjected to evaluate their α-amylase inhibition activity through the method documented by Xiao et al. (2006). Reaction mixture (100 μl) was composed of 30 μl PBS (50 mM), 10 μl enzyme (0.03 U/100 μl), 20 μl test sample and 40 μl of soluble starch (2 g/L). Plates were placed at 50°C for almost 30 min. To stop enzymatic reaction 20 μl of HCl (1 M) was pipetted in all reaction mixtures and at the end iodine reagent (100 μl; Iodine (5 mM) and potassium iodide (5 mM)), was added. The color change and absorbance was recorded at 540 nm. Inhibition percentage of the enzyme was measured by using following formula:

$$\begin{array}{cc} \%\;Relative\;enzyme\;activity & \\ & =(enzyme\;activityof\;test/enzyme\;activity\;of\;control)*100 \end{array}$$

(2) α−amylase inhibition(%)=(100−% relative enzyme activity).

IC_50_ values were calculated by using table curve software through linear regression analysis.

### DPP-4 Inhibition Assay

DPP-4 inhibition activity was assessed by using fluorometric technique, following the method documented by Saleem et al., 2014. This method calculates the quantity of free AMC (7-amino-4-methyl-coumarin). The AMC is released from the Gly-Pro-AMC which is the substrate of DPP-4. Experiment was performed in triplicate. Fluorescence was measured at Em 430 nm following excitation at Ex351 nm using a Tecan safire desktop fluorometer (Reading, England, United Kingdom). 50 mM HEPES buffer was used for preparation of each sample, whose pH was 7.4. To each well we added DPP-4 (20 μl 1 U/ml), test sample (20 μl) and 1 mM AMC (30 μl). Plates were placed at 37°C for 1 h and then 3 mM acetic acid (100 μl) was pipetted to stop the reaction. We used Berberine (13 mM), as a control for each experiment, as already reported (Al-Masri et al., 2009). IC_50_ for inhibition of DPP-4 was calculated for each sample.

### AMPK Activation Potential

To evaluate the AMPK activation potential of PA we performed *in vitro* analysis. We used primary hepatocytes, derived from C57BL/6 mice (Cell Biologics; Cat. C57-6224F). Hepatocytes were kept in M199 (Corning), supplemented with FBS (fetal bovine serum 10%) and glutagro (1% Corning). Hepatocytes were then serum starved, overnight, and prior to procedure in RPMI 1640 (United States Biological Life Sciences, R8999). 8 h prior to harvest, cells were treated with glucagon (2 μM), or test sample (20 μM). Cells were rinsed with ice cold PBS, twice, after harvest and were placed on ice until the extraction of protein was carried out. Assay was conducted in triplicate with three plates for each treatment. Protein from cells was hauled out; quantified and western blot was performed to assess the activation of AMPK after treatment with PA and compared it with untreated control and the cells treated with glucagon.

### Protein Extraction and Western Blot Analysis

Extraction of proteins and western blot analysis was carried out, as we already reported (Akhtar et al., 2016). Briefly, lysis buffer was added to the harvested cells. The composition of lysis buffer was 50 mM Tris–Hcl, 150 mM NaCl, 1 mM ethylene glycol-bis (aminoethylether)-tetraacetic acid, 1 m Methylene di amine tetra acetic acid, 20 mM NaF, 100 mM Na3VO4, 0.5% NP-40, 1% Triton X-100, 1 mM phenyl methyl sulfonyl fluoride, pH 7.4 with freshly added protease inhibitor cocktail (Protease Inhibitor Cocktail Set III, Calbiochem, La Jolla, CA). Plates were placed on ice for 30 min. Cells were scraped with the scraper and lysate was collected in eppendorf tube and passed through needle of the syringe to break up the cell aggregates. Lysate was cleared by centrifugation at 14000 × *g* for 30 min at 4°C and the supernatant (whole-cell lysate) was used or immediately stored at -80°C. Concentration of protein in each lysate was measured using Pierce BCA protein assay kit (Thermo Scientific), as per manufacturer’s protocol. For western blotting 4–12% poly acrylamide gels were used to resolve 30 μg of protein, transferred on to a nitrocellulose membrane, probed with appropriate monoclonal primary antibodies and detected by chemiluminescence autoradiography after incubation with specific secondary antibodies.

### Molecular Docking Studies

Molecular docking analysis was carried out using the software molecular docking analysis (MOE) and MOE 2018 was used, as reported by Ali et al. (2018). MOE is a licensed software system^1^ designed by the Chemical Computing Group to support cheminformatics, molecular docking, bioinformatics, virtual screening, and structure-based-design and can be used to build new applications based on scientific vector language (SVL). The structures of ligands were prepared using builder tool of MOE and then energy was minimized using force field MMFF94x and these structures with minimized energy were saved in the mdb file format. The 3-D structure of α-glucosidase, α-amylase and dipeptidyl peptidase 4 (DPP-4) were obtained from the protein data bank (PDB). The 3D structure of α-glucosidase [PDB 2ZE0] and α-amylase [PDB: ID1HNY] were obtained from the PDB^2^ Selected 2ZE0 and 1HNY were fixed with the force field MMFF94Xx to add up the hydrogen atoms, partial charges and missing residues that can be used properly for the processes of molecular docking.

### Development of HPLC Quantification Method

#### Instrumentation and Analytical Conditions

To select λ_max_ in different mobile phases for HPLC analysis 100 μg/ml PA solutions were subjected to spectrophotometer scan by using PDA spectrophotometer (Aligent; United States). For respective mobile phase different wavelengths were selected from the spectrum. Chromatographic analysis was carried out by using reverse phase high performance liquid chromatography, coupled with diode array detector (RP-HPLC-DAD). Zorbex RX-C8 analytical column (Agilent Tech; United States) was used for chromatographic separation. RP-HPLC-DAD conditions were optimized and mobile phase A, composed of MeOH:H_2_O/3:1, and mobile phase B, composed of MeOH (100%), were used. The mobile phase was freshly prepared, filtered and ultra-sonicated for 5 min before use. Initially isocratic 50% B was used for 0–15 min; then a gradient 15–18 min for 50% to 100% B; then again isocratic 100% B was used for 18–23 min.

### Standard Preparation

PA (10 mg) was dissolved in 5 ml of MeOH and sonicated for 5 min. The solution was diluted to 1 mg/ml and then serial dilutions were carried out to obtain 500, 250, 100, 50, 25, 10 and 2.5 μg/ml. Peak areas of eight concentrations (2.5–1000 μg/ml) were used to plot calibration curve of PA. Chromatogram was recorded thrice for each dilution.

### Sample Preparation

Stem, root, adventitious root (ad. root), leaf and fruit of *Ficus microcarpa* were collected. Each plant part was dried and almost 20 mg powder was extracted using 200 μl of MeOH/ Chloroform (1:1).

### Statistics

All experiments were performed in triplicate and mean values were calculated. IC_50_ values were calculated using table curve software. All statistical analysis was carried out with GraphPad prism (San Diego, CA).

## Results

### α-Glucosidase Inhibitory Activities

**FIGURE 4:**
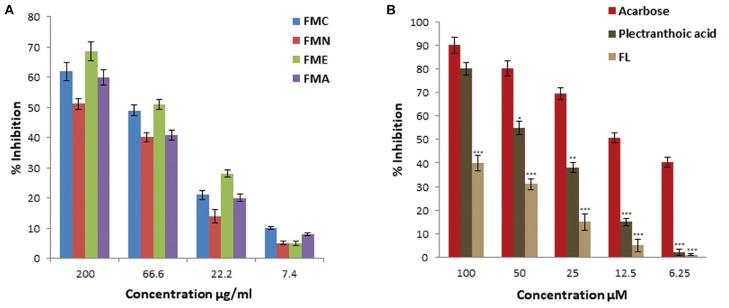
Percentage α-glucosidase inhibitory activity of crude extract, fractions and pure compounds from *Ficus microcarpa* **(A)**. Crude extracts of *Ficus microcarpa* and its fractions FMC; Crude extract of *Ficus microcarpa*, FMN; n-Hexane fraction, FME; Ethyl acetate fraction, FMA; Aqueous fraction. **(B)** PA and FL. Values are presented as mean ±*SD* (^∗^*P* ≤ 0.05, ^∗∗∗^*P* ≤ 0.001).

In current investigation, crude extract of *Ficus microcarpa* (methanol:chloroform/1:1); its fractions i.e., n-hexane (FMN), ethyl acetate (FME), aqueous (FMA) and the isolated compounds were subjected to evaluate α-glucosidase inhibition potential. Enzyme inhibition was calculated as percentage of inhibition as shown in Figure 4. Inhibitory activity of FMC and fractions varied noticeably. The α-glucosidase inhibitory activity of FMC, FMN, FME, and FMA was 62 ± 3%, 51.2 ± 1.8%, 68.6 ± 3.1%, and 60 ± 2.5% at 200 μg/ml, 49 ± 1.8%, 40.2 ± 1.5%, 51 ± 1.6%, and 40 ± 1.7% at 66.6 μg/ml, 21 ± 1.5%, 14 ± 2.2%, 28 ± 1.2%, and 20 ± 1.1% at 22.2 μg/ml, and 10 ± 0.5%, 5 ± 0.6%, 5 ± 0.8%, and 8 ± 0.4% at 7.4 μg/ml, respectively. The observed α-glucosidase inhibitory activity of crude extract and fractions in descending order was; ethyl acetate fraction (FME) > crude extract (FMC) > aqueous fraction (FMA) > n-hexane fraction (FMN), as shown in Figure 4A. All samples exhibited concentration dependent inhibition of α-glucosidase. IC_50_ values of FMC, FMN, FME, and FMA were 80.3 ± 5.4 μg/ml, 175.04 ± 6.5 μg/ml, 61.2 ± 5.5 μg/ml, and 135.9 ± 6.8 μg/ml, respectively. Among compounds (PA) showed better inhibitory activity as compared to 3,4,5,7- (FL), as shown in Figure 4B. IC_50_ values of PA and FL were found to be 39.5 ± 3.5 μM and 200 ± 6.8 μM, respectively. The IC_50_ value of acarbose was 12.5 ± 2.1 μM, used as positive control.

### α-Amylase Inhibitory Activities

**FIGURE 5:**
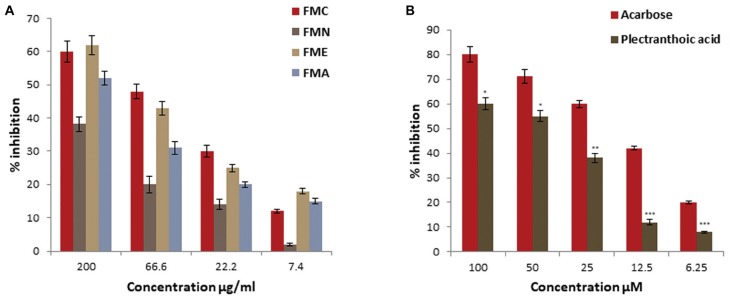
Percentage α-amylase inhibitory activity of crude extract, fractions and pure compound from *Ficus microcarpa* **(A)** crude extracts of *Ficus microcarpa* and its fractions FMC; crude extract, FMN; n-hexane fraction, FME; ethyl acetate fraction and FMA; aqueous fraction. **(B)** PA. Values are presented as mean ±*SD* (^∗^*P* ≤ 0.05, ^∗∗^*P* ≤ 0.01, and ^∗∗∗^*P* ≤ 0.001).

α-Amylase inhibitory activity of crude extract, fractions (FMN, FME, and FMA) and isolated compound (PA) was evaluated at different concentrations, as shown in Figure 5A. First crude extracts and fractions were subjected to α-amylase inhibition assay. FME showed better inhibitory activity, with percentage inhibition 62 ± 2.9%, 43 ± 2.1%, 25 ± 1.1%, 18 ± 0.9% at 200, 66.6, 22.2, and 7.4 μg/ml, respectively, (Figure 5A), as compared to other fractions. Inhibitory activity of crude extract and fractions in the descending order was FMC > FME > FMA > FMN, with IC_50_ values 77 ± 2.5 μg/ml, 85.2 ± 5.5 μg/ml, 186 ± 5.5 μg/ml, and > 200 μg/ml, respectively. Due to potent inhibition activity, FME was selected for isolation of bioactive compounds with anti-hyperglycemic potential. PA was subjected to α-amylase inhibitory activity and results showed that PA exhibited significant α-amylase inhibition activity in a concentration dependent manner. The percentage of inhibition was 60 ± 2.5%, 55 ± 2.1%, 38 ± 1.8%, 12 ± 1.1%, and 8 ± 0.5% at 100, 50, 25, 12.5, and 6.25 μM, respectively, with IC_50_ value 55.5 ± 2.5 μM (Figure 5B). FL was not included in this study because of its low efficacy in inhibiting α-glucosidase. Acarbose was used as positive control for α-amylase inhibition with IC_50_ value 16.6 ± 3.5 μM.

### Dipeptidyl Peptidase-4 Inhibition

**FIGURE 6:**
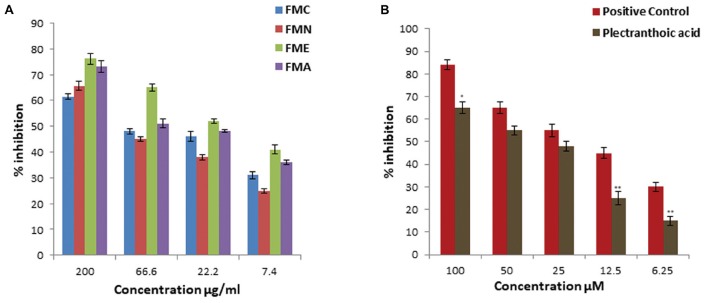
DPP-4 inhibition activity of crude extract, fractions and pure compound from *Ficus microcarpa* **(A)** Crude extract of *Ficus microcarpa* and its fractions. FMC; *Ficus microcarpa* crude extract, FMN; n-hexane fraction, FME; ethyl acetate fraction and FMA; aqueous fraction. **(B)** PA. Values are presented as mean ±*SD*. (^∗^*P* ≤ 0.05, ^∗∗∗^*P* ≤ 0.001).

We assessed DPP-4 inhibition activity of crude extract and fractions of *Ficus microcarpa*. Results showed that DPP-4 inhibition potential of crude extract (FMC) and fractions varied significantly might be due to difference in the composition of compounds within each sample. The inhibitory activity of crude extract was 61.5 ± 1.2%, 65.6 ± 1.8%, 76.1 ± 2.2%, and 73.1 ± 2.3% at 200 μg/ml as shown in Figure 6A. Among fractions, FME showed maximum inhibitory activity with percentage of inhibition 76.1 ± 2.2%, 65 ± 1.5%, 52 ± 0.9%, and 41 ± 1.8% at 200, 66.6, 22.2, and 7.4 μg/ml, respectively. Calculated IC_50_ values of FMC and fractions in descending order were FME (20.5 ± 2.3 μg/ml) > FMA (62 ± 1.3 μg/ml) > FMC (95.2 ± 2.1 μg/ml) > FMN (109.5 ± 5.6 μg/ml). PA showed significant DPP-4 inhibition activity with percentage of inhibition 65 ± 2.6%, 55 ± 2.1%, 48 ± 2%, 25 ± 2.9%, and 15 ± 2% at 100, 50, 25, 12.5, and 6.25 μM, respectively, and IC_50_ value of PA was 51.4 ± 2.0 μM (Figure 6B). Berberine was used as the positive control for DPP-4 inhibition and with IC_50_ value 27.4 ± 2.2 μM.

### AMPK Activation Potential

**FIGURE 7:**
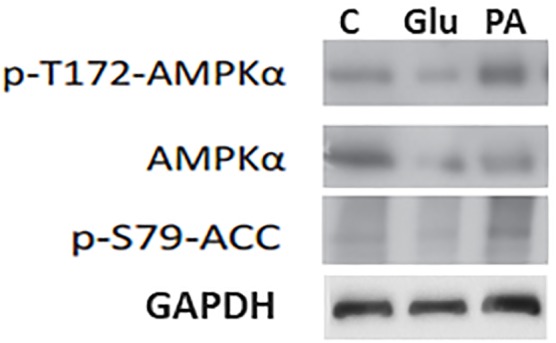
Induction of p-AMPK and p-ACC by PA. PA induced the phorphrylation of AMPK in primary hepatocytes. To confirm equal loading blots were reprobed with loading control i.e., GAPDH. p-AMPK; Phospho 5′ AMP-activated protein kinase, ACC; Acetyl-CoA carboxylase, C; Control vehicle only, PA; PA, Glu; Glucagon.

Most of the drugs with antidiabetic potential, such as metformin, activate AMPK. As we found that PA had anti-hyperglycemic potential we further assessed the AMPK activation potential of PA, using primary hepatocytes. For this purpose we treated the cells with PA (20 μM). Results showed that PA activates AMPK by phosphorylation AMPK at Th^172^, as shown in Figure 7. Glucagon was used as a control because it increase inhibitory phosphorylation of AMPK (Ser^173^) and reduce active phosphorylation of AMPK (Thr^172^) due to PKA-mediated inhibition of AMPK (Aw et al., 2014). We observed that there was no AMPK activation in cells treated with glucagon and vehicle control, as expected. Further, we evaluated the effect of PA mediated AMPK activation on phosphorylation of acetyl-CoA carboxylase (ACC). Results from the western blot showed that AMPK was activated by PA which in turn phosphorylated ACC to p-ACC at S^79^ site (Figure 7). The phosphorylation was not observed in glucagon and vehicle control.

### Molecular Docking

**FIGURE 8:**
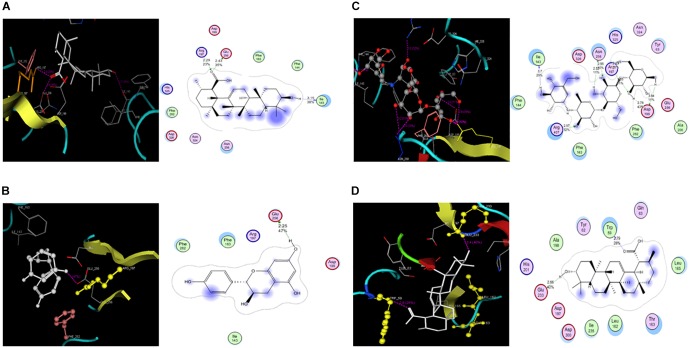
Molecular docking PA, FL and Acarbose **(A)**. PA docked with α-glucosidase **(B)**. Docked FL **(C)**. Acarbose in the active site **(D)**. Docked poses of PA and Acarbose in active site of α-amylase.

Molecular docking studies of PA, FL and acarbose were performed in the active site of α-glucosidase [PDB 2ZE0] and α-amylase [PDB ID 1HNY]. Binding energies of these compounds are given in Table 1, 2. Docking score of PA with α-glucosidase was -11.5 while with α-amylase was -5.63. FL was also docked with α-glucosidase and recorded binding energy was -9.13. Docking poses and binding patterns of the pure compounds and acarbose, used as standard, are shown in Figure 8.

**Table 1:** Binding energies of docked compounds with α-glucosidase and α-amylase.

Compound	α-Glucosidase		α-Amylase	
		
	IC_50_	Dock Score	IC_50_	Dock Score
PA	39.5	-11.52	55.5	-5.63
FL	200	-9.13	NT	–
Acarbose	12.5	-12.82	16.6	-9.72


**Table 2:** Analysis of the ligand interactions with localized amino acids residues at binding sites of α-glucosidase and α-amylase.

Compound	α-Glucosidase		α-Amylase	
		
	H-bonding	AA	H-bonding	AA
PA	2.29, 2.43, 3.15	Arg197, Glu256, Ile143,	2.59, 2.66	Trp59, Glu233
FL	2.25	Glu256	NT	-
Acarbose	(2.95,2.52), 3.7, (2.78, 3.84),2.83	Asn258, Ile143, Asp199, Arg197	1.22, 1.99	Trp59, Glu233


### Quantification of PA in Different Parts of *Ficus microcarpa*

**FIGURE 9:**
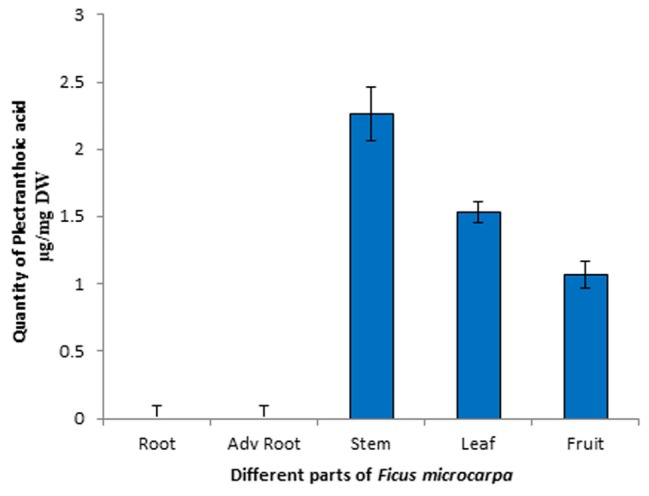
High-performance liquid chromatography (HPLC) profile of PA in different parts of *Ficus microcarpa*. Quantification of PA in different parts of *Ficus microcarpa*. Adv root, adventitious root. DW, Dry weight. Significant differences between the amount of PA were observed (*p* < 0.001) in each part of *Ficus microcarpa*.

Quantitative analysis of PA in different parts of *Ficus microcarpa* i.e., root, stem, leaf and adventitious roots was carried out by using reverse phase HPLC. Chromatographic profile was compared with the retention time and absorption spectrum of reference standard. Calibration curve of the standard compound was constructed and its statistical analysis is shown in Table 3. Representative chromatograms of PA in stem and leaf are shown in Supplementary Figure 3 while of fruit, adv. root and root are shown in Supplementary Figure 4. The results revealed that maximum amount of PA was present in stem i.e., 2.2 ± 0.2 μg/mg dry weight (DW), while in leaf and fruit 1.529 ± 0.08 μg/mg DW and 1.06 ± 0.1 μg/mg DW quantity was detected, respectively. Moreover, the results revealed that PA was not found in roots and adventitious roots of *Ficus microcarpa*. Results are presented in Figure 9.

**Table 3:** Statistical analysis of calibration curve of Plectranthoic acid.

Studied Parameters	Results
Beer’s law limit	2.50–1000
λ_max_	210 nm
Regression Equation	*y* = 1.3697 *x* - 13.10
Correlation coefficient	0.9997
Detection limit	2.5 μg/ml
Quantification limit	2.5 μg/ml


## Discussion

From previous studies in which we screened 61 medicinal plants (Akhtar et al., 2015), we concluded that *Ficus microcarpa* is one of the most promising plants with therapeutic potential for further studies due to high total phenolic and total flavonoid content. As former studies using albino wistar rats reported that *Ficus microcarpa* possess anti-diabetic activity (Lakshmi et al., 2010), we hypothesized that the isolated compounds might have such properties. Type 2 diabetes (T2DM) is the 7th leading cause contributing to death of many individuals in United States and current therapies leave much room for improvement. Most of the suffered individuals require multiple prescriptions to reduce glucose levels of blood (Tiwari and Rao, 2002). In proposed study, we assessed the anti-hyperglycemic potential of *Ficus microcarpa* and isolated pure compounds, evaluated their potential use against T2DM. We collected aerial parts of *Ficus microcarpa* and prepared crude extract through maceration process using M/C (1:1). The FMC was then subdivided into different fractions by solvent-solvent extraction i.e., non- polar n-hexane fraction (FMN), moderately polar ethyl acetate fraction (FME) and highly polar aqueous fraction (FMA). Crude extract and fractions were then subjected to α-glucosidase, α-amylase and DPP4 inhibition assays. Previous studies showed that inhibition of just α-glucosidase significantly reduces glucose levels and clinically it is used extensively for treatment of T2DM (Etxeberria et al., 2012). Current research revealed that *Ficus microcarpa* is a valuable source of natural compounds which can inhibit α-glucosidase, α-amylase and DPP-4, consequently, can be used for T2DM treatment. The results correlate with the study of Lakshmi et al. (2010) whose work suggests that *Ficus microcarpa* might be new clinical choice in (DM) treatment.

Our results showed that FME had better α-glucosidase and α-amylase inhibition as compared to other fractions. Interestingly, other fractions also showed good percentage of inhibition for above mentioned enzymes. This suggests that *Ficus microcarpa* is a rich source of bioactive compounds of diverse polarity having anti-hyperglycemic potential. Usually phenolic compounds possess strong inhibitory activity possibly due to the presence of the glycosidic groups on phenolic scaffolds (Apostolidis et al., 2007). Recently, the DPP-4 enzyme is going to be famous and has emerged as important target of antidiabetic drugs. Pharmaceutical industries have made efforts which resulted in the development and improvement of DPP-4 inhibitors; having better safety and efficacy profiles. Unfortunately, there are few studies about the presence of DPP-4 inhibitors in medicinal plants (Parmar et al., 2012). In presented work, we evaluated the DPP-4 inhibition potential of *Ficus microcarpa* and it is worthy to mention that *Ficus microcarpa* possessed significant ability to inhibit DPP-4. Maximum inhibition percentage was exhibited by FME with IC_50_ 20.5 ± 2.3 μg/ml.

Next, we designed experiments to isolate compounds with anti-hyperglycemic potential Based on the results of the assays; FME was selected for isolation of bioactive compounds. Bioactivity guided isolation yielded 2 pure compounds, 1). (PA); 2). 3,4,5,7- (FL). PA is a pentacyclic triterpenoid. Triterpene is a diverse class of natural compounds and several diverse structures of these are reported until now. Pentacyclic triterpenes comprised of 30-carbon skeleton with five or four six-membered rings and a five-membered ring. PA was first isolated by Razdan and his colleagues from *Plectranthus rugosus* in 1982. We have isolated this compound from *Ficus microcarpa* for the first time. Other isolated compound was FL, which is a flavonoid and literature showed that flavonoids have important effects on cancer chemoprevention and chemotherapy (Chahar et al., 2011). FL/Afzelechin is a flavan-3-ol, reported to be isolated from rhizomes of *Bergenia ligulata* (Saijyo et al., 2008). Anti-hyperglycemic activities of flavonoids have been previously reported (Yan et al., 2014). These are also well known for DPP-4 inhibition potential (Parmar et al., 2012).

Among two compounds PA exhibited promising inhibitory activity against α-glucosidase and α-amylase at various concentrations as compared to FL. Hydroxyl (OH) groups are postulated to interact with yeast α-glucosidase active site amino acids by hydrogen bonds, thus inhibiting the catalysis of the substrate (Xu, 2010). Considering the structure of PA, it can be deduced that its OH group may interact with the enzyme active site and thus, is responsible for the inhibition of the enzyme activity. PA also showed DPP-4 inhibition, with IC_50_ 51.4 ± 2.0 μM, supporting its promising potential as anti-hyperglycemic agent. Many triterpenoids are already reported to have antidiabetic properties such as oleanolic acid and ursolic acid (Ali et al., 2006), but this is the first instance that the triterpenoid, i.e., PA, has been proposed to have anti-diabetic effects.

Recently, AMPK has emerged as an effective target for T2DM treatment, because it is the major regulator of energy within the cell. It is stimulated in calorie restriction and physical activities, thus increasing insulin sensitivity of tissues and enhancing glucose uptake. Pharmacological stimulation of AMPK provokes many benefits like improvement of insulin sensitivity and glucose homeostasis, making it an attractive target for T2DM and metabolic syndrome (Ruderman and Prentki, 2004). Although the drugs that activate AMPK are available i.e., Metformin, TZDs, but their potential for direct activation of AMPK is debatable, as many off-targets effects of these drugs are reported (Ben Sahra et al., 2011). There is a need of new AMPK activators with no side effects. In this study we evaluated the AMPK activation potential of PA using primary hepatocytes and found that PA is a potent activator of AMPK, which we already evaluated using cancer cells (Akhtar et al., 2016). We also presented that PA is non-toxic to normal cells (Akhtar et al., 2016). AMPK is reported as a regulator of ACC (acetyl-CoA). It phosphorylates ACC at serine residues including Ser^79^ (Park et al., 2002), thus playing role in regulation of fatty acid synthesis and degradation. To confirm activation of AMPK by PA, we further assessed the phosphorylation of ACC at Ser^79^. Results showed that PA treatment resulted in the phosphorylation of ACC which is the substrate of AMPK, thus confirms the role of PA as activator of AMPK.

*In silico* studies showed a good correlation with experimental results. Observed high activity of PA was might be due to its strong binding interactions with target. PA has strong hydrogen binding with Arg197 at distance of 2.29, Glu 256 at distance of 2.43 and Ile143 at distance of 3.15 (Figure 8A). Low activity of FL against α-glucosidase was might be due to its fewer efficacies with target as it has one hydrogen binding with Glu 256, as shown in Figure 8B. Acarbose has strong binding interaction with both α-glucosidase and α-amylase as shown in Table 2 and Figure 9C. Similarly, activity of PA against α-amylase was confirmed by its hydrophilic interactions with Trp 59 and Glu 233 as shown in Figure 8D. PA showed significant interactions and good binding scores with both targets (Figure 8), which increase its value as a potential dual inhibitor.

Next, we quantified PA in different parts of *Ficus microcarpa*. For this purpose, different parts of *Ficus microcarpa* i.e., root, adventitious root, stem, leaf and fruit were extracted by using different solvents among which n-hexane: ethyl acetate /2:1 was found to be most suitable with 98% recovery. This displayed greater selectivity for the analyte while reduced polar interfering compounds. Results revealed that maximum concentration of PA was perceived in stem while fewer in leaf and fruit. Moreover, HPLC results showed that PA is not found in roots and adventitious roots of *Ficus microcarpa*, so we recommend stem for the isolation of this potent compound.

In this study we proposed that PA is a potent compound with anti-hyperglycemic potential. *In-vivo* studies using animal models or diabetic patients, for detailed antidiabetic effects along with identification of potential targets, are recommended and are part of our future studies.

## Conclusion

Taken together study revealed that *Ficus microcarpa* is a valuable source of compounds with anti-hyperglycemic potential. Our studies showed that PA is a bioactive compound, the potential anti-hyperglycemic activities of which have hitherto remained unexplored. PA has potential to inhibit α-glucosidase, α-amylase and DPP-4 and is a potent activator of AMPK, thus can be used for the development of nutraceuticals and functional foods for T2DM. Evaluation of potent activities and identification of potential targets of PA in other clinical models, both *in vitro* and *in vivo*, is mandatory for its translation as an effective agent against T2DM.

## Author Contributions

NA was the major contributor of the presented work. LJ guided her in Bio activity guided isolation of compounds. BG helped for cell line assays and DPP IV inhibition assay. RA helped for NMR and structural analysis of the isolated compounds. SK worked on docking studies. This work was performed in the guidance and lab of BM.

## Conflict of Interest Statement

The authors declare that the research was conducted in the absence of any commercial or financial relationships that could be construed as a potential conflict of interest.

## Footnotes

1 https://www.chemcomp.com/MOE-Molecular_Operating_Environment.htm

2 http://www.rcsb.org/pdb

## Supplementary Material

FIGURE S1 NMR spectrum of Plectranthoic acid (PA): **(A)**. ^1^H NMR spectrum of PA **(B)**. ^13^C NMR spectrum of PA **(C)**. HMBC spectrum of PA **(D)**. HSQC spectrum of PA **(E)**. NOESY spectrum of PA **(F)**. COSY spectrum of PA.

FIGURE S2 NMR spectrum of 3,4,5,7-Flavantetrol/Afzelechin (FL) **(A)**. ^1^H NMR spectrum of FL **(B)**. ^13^C NMR spectrum of FL **(C)**. HMBC of FL **(D)**. HSQC of FL **(E)**. NOESY of FL **(F)**. COSY of FL.

FIGURE S3 Representative HPLC chromatograms of **(A)**. Standard **(B)**. Stem **(C)**. Leaf.

FIGURE S4 Representative HPLC chromatograms of **(A)**. Fruit **(B)**. Advantageous root **(C)**. Root.
