# Corrigendum: A Multi-Mode Bioactive Agent Isolated From *Ficus microcarpa* L. Fill. With Therapeutic Potential for Type 2 Diabetes Mellitus

**DOI:** 10.3389/fphar.2019.00643

**Published:** 2019-06-13

**Authors:** Nosheen Akhtar, Laila Jafri, Brian D. Green, Saima Kalsoom, Bushra Mirza

**Affiliations:** ^1^Department of Molecular Medicine, National University of Medical Sciences, Rawalpindi, Pakistan; ^2^Department of Biochemistry, Bahauddin Zakariya University, Multan, Pakistan; ^3^Advanced ASSET Centre, Institute for Global Food Security, School of Biological Sciences, Queen’s University Belfast, Belfast, United Kingdom; ^4^Pakistan Institute of Engineering and Applied Sciences, Nilore, Pakistan; ^5^Department of Biochemistry, Quaid-i-Azam University, Islamabad, Pakistan

**Keywords:** α-glucosidase, amylase, DPP4 (dipeptidyl peptidase-4), Ficus microcarpa, plectranthoic acid, AMPK

In the original article, there was an error. Throughout the manuscript, all instances of “Plectranthoic acid A (PA-A)” have been replaced with “Plectranthoic acid (PA)”.

The authors apologize for this error and state that this does not change the scientific conclusions of the article in any way. The original article has been updated.

