# Supplementary figures and images for: A Multi-Mode Bioactive Agent Isolated From Ficus microcarpa L. Fill. With Therapeutic Potential for Type 2 Diabetes Mellitus
Source: Front Pharmacol. 2018 Nov 27;9:1376. doi: 10.3389/fphar.2018.01376 (PMC6277780; doi:10.3389/fphar.2018.01376)

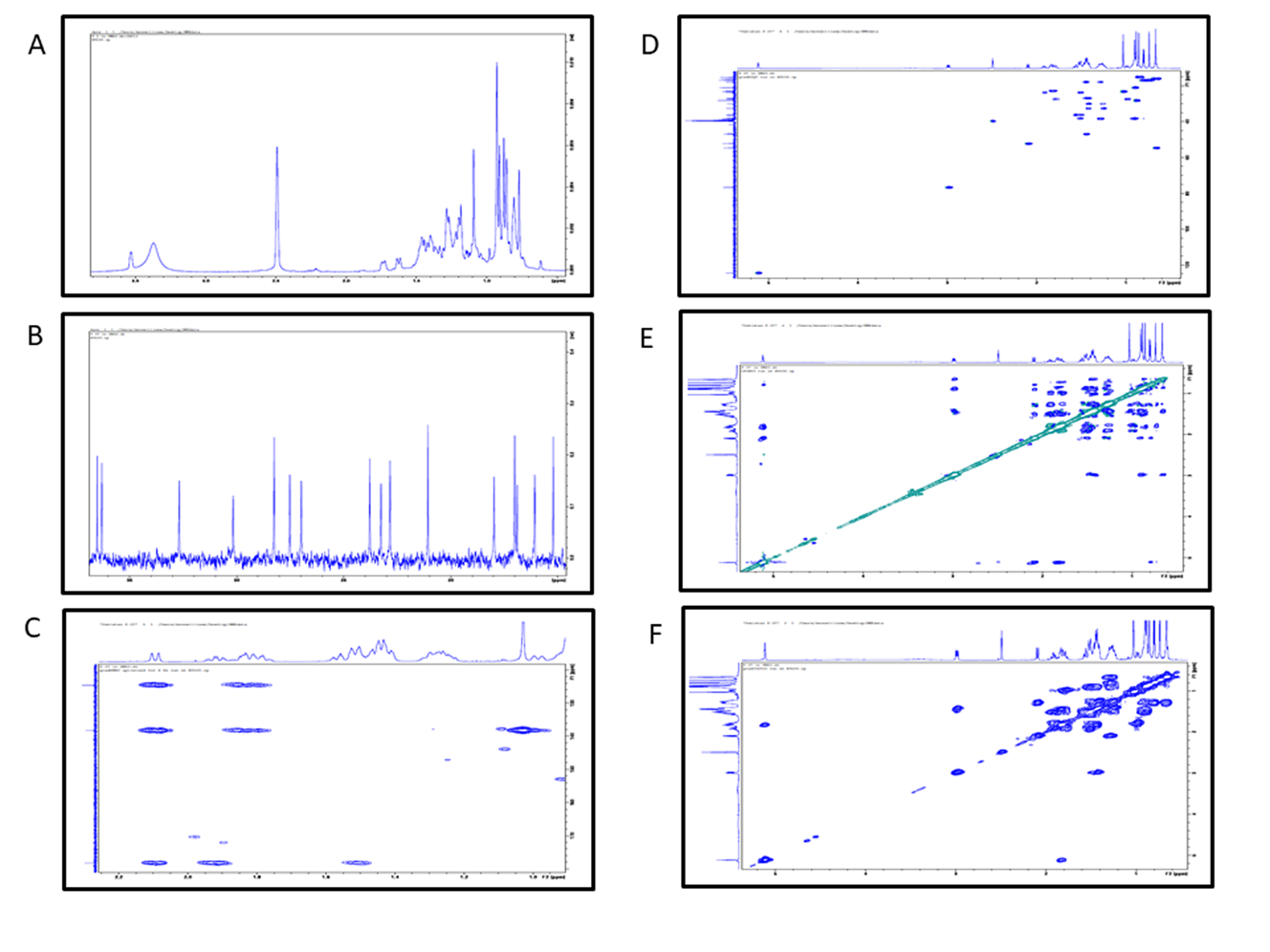

Supplement: FIGURE S1 — NMR spectrum of Plectranthoic acid (PA): (A). 1H NMR spectrum of PA (B). 13C NMR spectrum of PA (C). HMBC spectrum of PA (D). HSQC spectrum of PA (E). NOESY spectrum of PA (F). COSY spectrum of PA. [file Image_1.TIF]

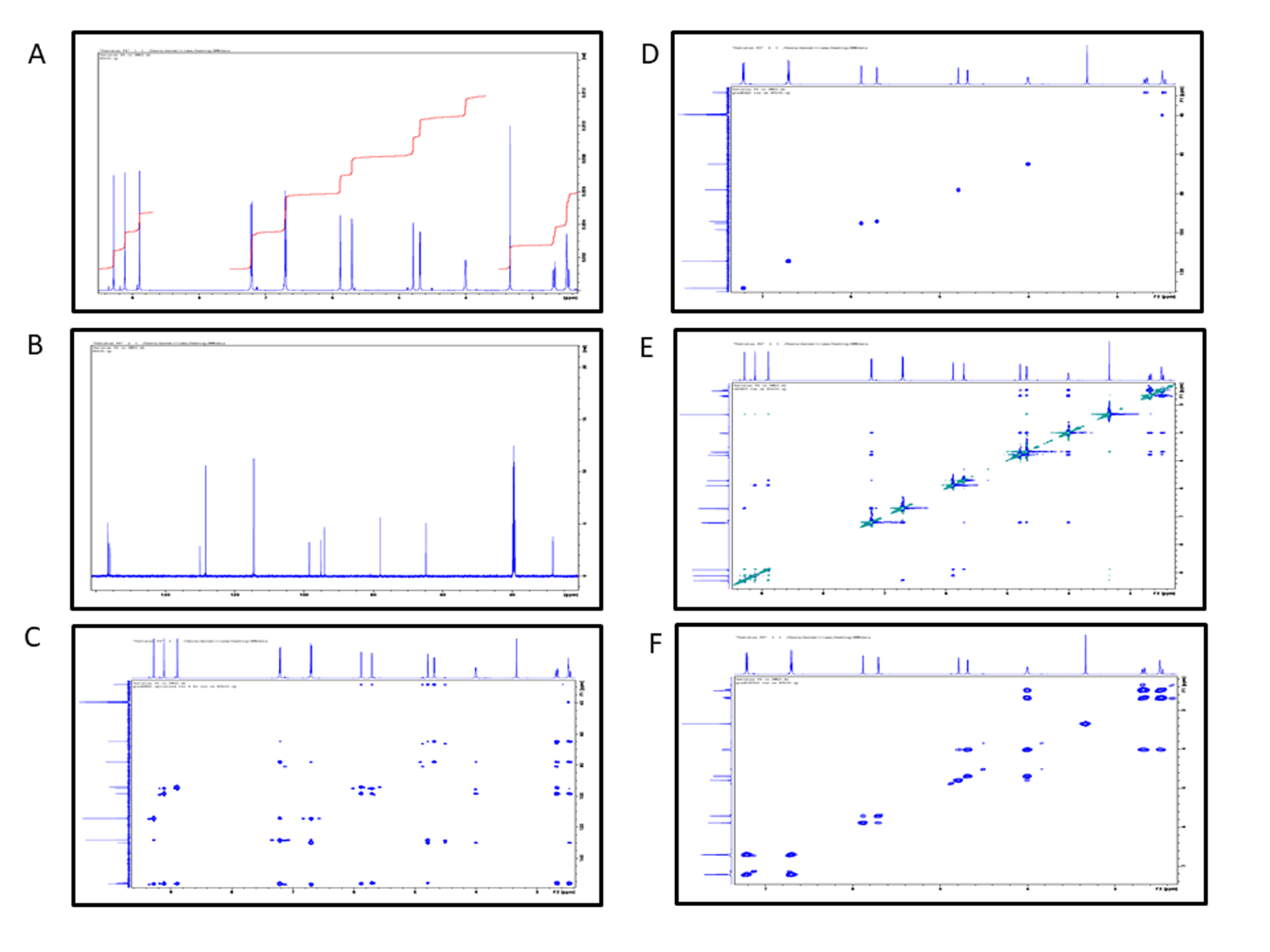

Supplement: FIGURE S2 — NMR spectrum of 3,4,5,7-Flavantetrol/Afzelechin (FL) (A). 1H NMR spectrum of FL (B). 13C NMR spectrum of FL (C). HMBC of FL (D). HSQC of FL (E). NOESY of FL (F). COSY of FL. [file Image_2.TIF]

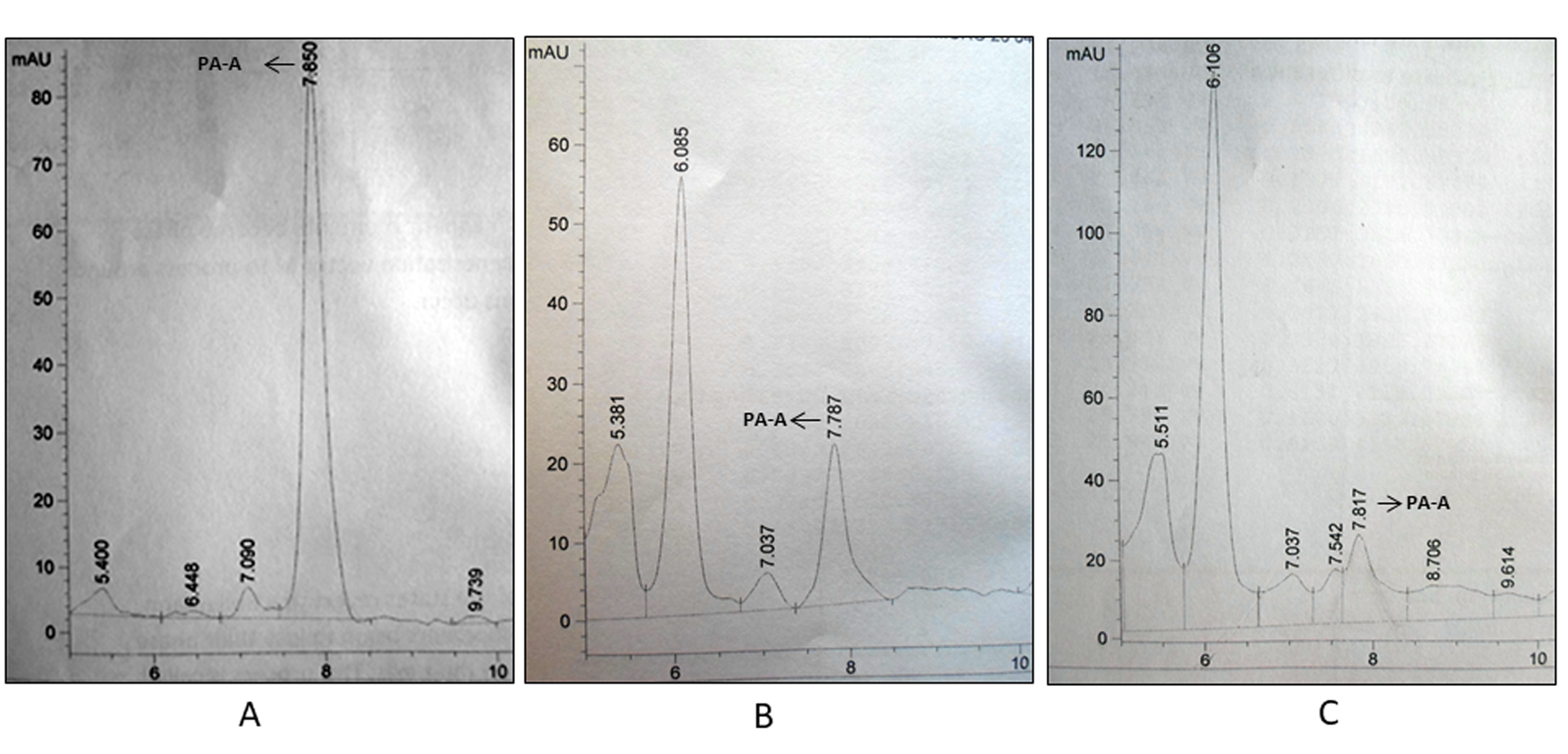

Supplement: FIGURE S3 — Representative HPLC chromatograms of (A). Standard (B). Stem (C). Leaf. [file Image_3.TIF]

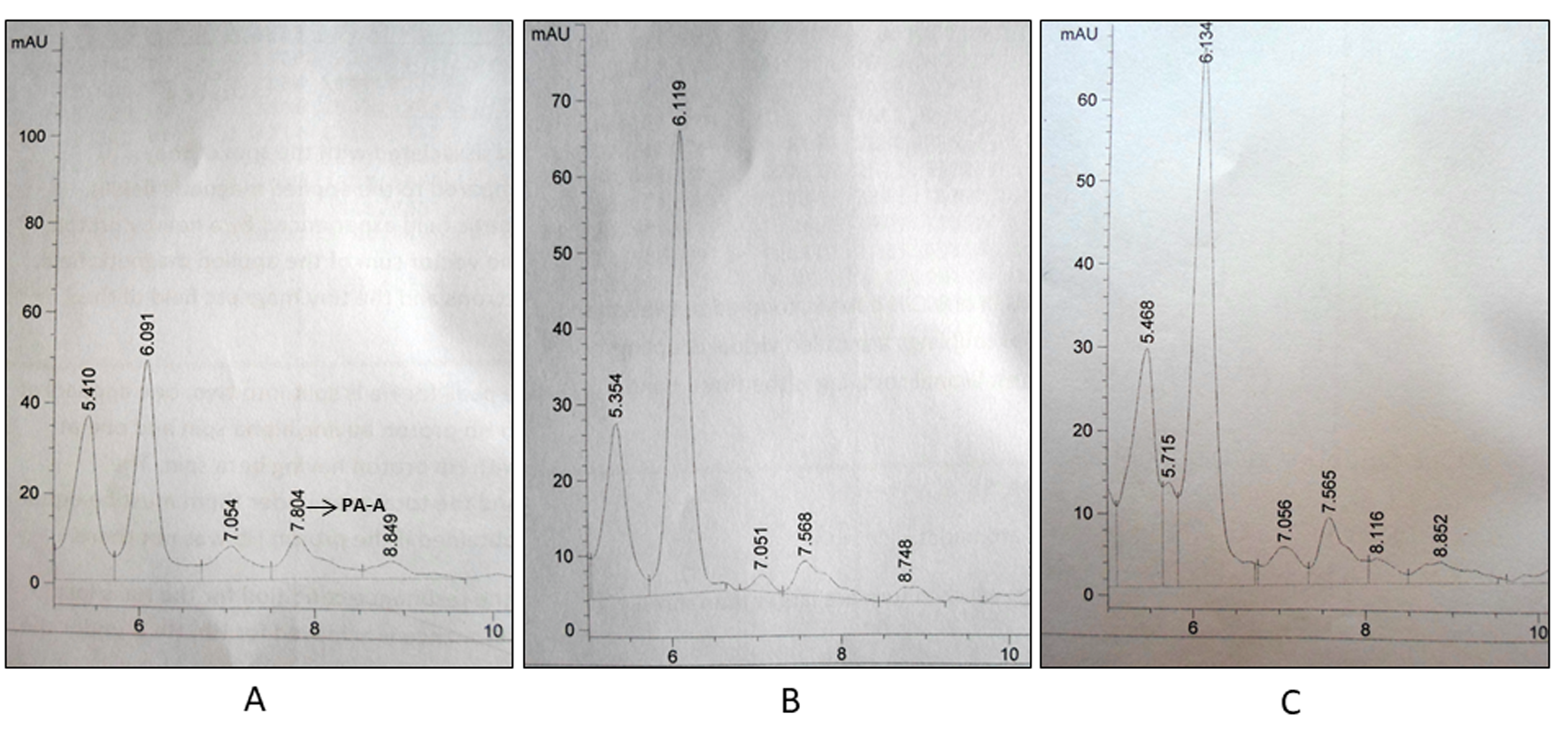

Supplement: FIGURE S4 — Representative HPLC chromatograms of (A). Fruit (B). Advantageous root (C). Root. [file Image_4.TIF]
